# Defying the Downtrend: Factors Driving Medical Students to Pursue Emergency Medicine

**DOI:** 10.1002/aet2.70057

**Published:** 2025-06-12

**Authors:** Danielle Kerrigan, Anita Knopov, Kaitlin Lipner, Lauren Trembley, Amy Mariorenzi, Brian Clyne

**Affiliations:** ^1^ Department of Emergency Medicine Alpert Medical School of Brown University Providence Rhode Island USA; ^2^ Department of Emergency Medicine University of California San Francisco Fresno California USA

## Abstract

**Background:**

In recent years, the number of medical students applying to Emergency Medicine (EM) has sharply decreased. Burnout and workforce projections within the specialty have been cited as contributing factors. Counter to this trend, our institution observed a higher percentage of students pursuing EM than the national average each year. This study sought to understand the factors driving these students to pursue EM.

**Methods:**

We performed a cross‐sectional survey of medical students from our institution who matched into EM from 2022 to 2024. Participants completed an electronic survey about influences on how they learned about EM and on their decision to pursue an EM career. The survey consisted of rating scale and short answer questions. A test of binomial proportions assessed differences between rating scale items using the item with the most positive results as the reference. Short answer responses were grouped into categories and ranked by frequency.

**Results:**

A total of 31 of 41 (76%) students responded. The factors found to be most useful for learning about EM as a specialty were “Participation in clinical electives” (*n* = 28, 93.3%, reference) and “Advising/mentoring from residents” (*n* = 27, 90%, *p* = 0.64). In terms of choosing EM, clinical exposure to the specialty, particularly within the fourth year (*n* = 28, 90.3%, reference), and advising from attendings (*n* = 23, 74.2%, *p* = 0.1) and residents (*n* = 25, 80.6%, *p* = 0.28) were among the most influential factors. Most students decided to pursue EM during their third year (65%, *n* = 15).

**Conclusion:**

The most influential factors driving our medical students to pursue EM were clinical exposure to EM and individual advising, particularly from EM residents. Focused efforts in these areas are potential strategies for other institutions to foster student interest in EM and counter the national trend.

## 1 Introduction

The number of senior medical students applying into US‐based Emergency Medicine (EM) residencies declined over 2022–2023. Concomitantly, the number of unfilled EM residency spots in the National Residency Match Program (NRMP) had increased, with 217 unfilled spots in 2022 and 554 unfilled spots in 2023 [1]. The data suggest a decline in student interest in EM and have spurred discussions about the impact of burnout, the COVID‐19 pandemic, and clinical stressors on the specialty [2, 3, 4, 5, 6, 7]. Workforce projections and concern over an oversupply of EM physicians may have also contributed to student interest [7, 8, 9, 10].

Despite the downturn, our institution consistently observed a higher percentage of students selecting EM than the national average. We saw an increase in applicants in 2023 (12.8%, from 8.2% in 2022), while the national average decreased from 5.7% to 4.5% over the same period [1]. While we had a slight decrease in applicants in 2024 (10.7%), this was still far greater than the national average of 4.1% [1]. This study sought to examine our students' divergence from the national trend.

Our institution offers an elective clerkship for third and fourth year students as well as an Advanced Clinical Mentorship (ACM) elective for third year students in which they work directly with an EM faculty mentor on shift. Fourth year students applying to EM take a separate, required sub‐internship, which includes more intensive clinical hours and didactics. Pre‐clinical students are invited to participate in Emergency Medicine Interest Group (EMIG)‐sponsored events and shadow resident physicians.

We recently established a Resident Student Education Committee (RSEC) as a novel, structured approach to support student education and advising within EM [11]. The RSEC is led by residents who provide longitudinal outreach to students through EMIG activities, advising workshops, shadowing experiences, and individualized mentoring. All students participating in the sub‐internship are assigned an RSEC mentor, while the other RSEC activities are optional for students. Faculty also host a series of informal group mentoring meetings for students interested in EM throughout the year that discuss various topics, including away rotations, interviews, and creating a rank list. Students applying into EM are formally assigned an individual EM faculty mentor at the beginning of their fourth year to assist with the application process.

Numerous factors have been shown to influence the decision to pursue a career in EM, ranging from lifestyle factors to the clinical environment to early exposure through pre‐hospital care or third year clerkships [12, 13, 14, 15, 16]. This study sought to understand the factors influencing students at our institution over a specific period. We sought to determine the experiences influencing: (1) how students learned about EM, and (2) how they chose EM as a career.

## 2 Methods

All medical students from our institution who matched into EM through the NRMP Main Residency Match from 2022 to 2023, and those applying to match in 2024, were eligible to participate. Students who matched into EM through the Supplemental Offer and Acceptance Program (SOAP) process were excluded. Eligible students received an email with a link to an anonymous electronic Qualtrics survey in November 2023, followed by two reminder emails between November and December 2023. The study was considered exempt by our Institutional Review Board.

The survey (Appendix A) included rating scale questions about the usefulness of various resources at our institution in terms of: (1) learning about EM as a specialty and (2) influencing the decision to pursue EM. Responses of “very useful/influential” and “moderately useful/influential” were combined; and responses of “somewhat useful/influential” and “not at all useful/influential” were combined to form two groups. Responses of “did not use” were omitted. A test of binomial proportions was conducted to assess the differences between items using the item with the most positive results as the reference. This methodology allowed us to dichotomize responses and assess the proportion of usefulness of each item.

Short answer questions allowed students to describe the most influential factors for pursuing EM. These responses were grouped into categories, then ranked according to response frequency. Responses were further categorized into “non‐modifiable” factors, which are those intrinsic to the field of EM, and “modifiable” factors, which are variable and can be influenced at the medical school level, to identify potential programming opportunities. Additional short answer questions asked about students' first exposure to EM and when they ultimately decided to pursue EM.

## 3 Results

A total of 31 of 41 (76%) eligible students completed the survey. Responses to multiple choice questions are listed in Table 1.

**TABLE 1:** Survey results.

	Useful/influential factors	Less useful/influential factors
Learning about Emergency Medicine as a specialty	Participation in clinical electives^a^ (*n* = 28, 93.3%) Advising/mentoring from residents (*Z* = 0.47, *p* = 0.64)	Advising/mentoring from attending physicians (*Z* = 2.34, *p* < 0.05) School‐sponsored career planning workshops and courses (*Z* = 3.65, *p* < 0.001) Emergency medicine‐sponsored career planning workshops, meetings, or lectures (*Z* = 2.28, *p* < 0.05) Exposure to EM via the interest group (*Z* = 3.0, *p* < 0.01)
Choosing Emergency Medicine as a specialty	Exposure during a 4th year clerkship or sub‐internship^a^ (*n* = 28, 90.4%) Advising/mentoring from attending physicians (*Z* = 1.66, *p* = 0.1) Advising/mentoring from residents (*Z* = 1.08, *p* = 0.28) Exposure to EM during 1st or 2nd year of medical school (*Z* = 1.36, *p* = 0.17) Exposure to EM during 3rd year clerkship (*Z* = −0.02, *p* = 0.99) Exposure to EM during 3rd year elective (*Z* = 0.10, *p* = 0.92) Resident‐led teaching (*Z* = 1.57, *p* = 0.12)	Exposure to emergency medical care prior to starting medical school (*Z* = 2.85, *p* < 0.01) Research opportunities within EM (*Z* = 4.5, *p* < 0.01) Faculty‐led teaching (*Z* = 2.59, *P* < 0.01) Personal experience or a friend/family member receiving emergency care (*Z* = 4.01, *p* < 0.001)

^a^ Reference group.

In terms of learning about EM, the item with the most positive result and therefore the reference group, was “Participation in clinical electives” (*n* = 28, 93.3%). “Advising/mentoring from residents” was not significantly different from the reference group (*n* = 27, 90%, *p* = 0.64), suggesting that it is as useful for learning about EM.

Less useful factors for learning about EM included “Advising/mentoring from attending physicians,” “School‐sponsored career planning workshops and courses,” “EM‐sponsored career planning workshops, meetings, or lectures,” and “Exposure to EM via the interest group.”

In terms of choosing EM as a specialty, “Exposure during a fourth year clerkship or sub‐internship” (*n* = 28, 90.3%) had the most positive response and was therefore made the reference group. Factors found to be as influential included: “Advising/mentoring from attending physicians” (*n* = 23, 74.2%, *p* = 0.1), “Advising/mentoring from residents” (*n* = 25, 80.6%, *p* = 0.28), “Exposure to EM during 1st or 2nd year of medical school” (*n* = 20, 76.9%, *p* = 0.17), “Exposure to EM during 3rd year clerkship” (*n* = 19, 90.5%, *p* = 0.99), “Exposure to EM during 3rd year EM Elective” (*n* = 17, 89.5%, *p* = 0.92) and “Resident‐led teaching” (*n* = 21, 75%, *p* = 0.12).

Factors found to be less influential for choosing EM were: “Exposure to EM prior to starting medical school,” “Research opportunities within EM,” “Faculty‐led teaching,” and “Personal experiences with a friend/family member receiving emergency care.”

Responses to the open‐ended question pertaining to their top 3 most influential factors in choosing EM are listed in Table 2. The most common responses included the non‐modifiable factors “Scope of practice/the medicine,” “Lifestyle/work‐life balance,” and “People/culture.”

**TABLE 2:** Top three most influential factors in choosing EM.

Factor	# Times ranked in top 3	# Times ranked most influential
Scope of practice/the medicine	14	6
Lifestyle/work–life balance	12	1
People/culture	8	5
Clinical exposure MS3/MS4^a^	5	3
Resident mentorship^a^	5	2
SEM/advocacy	5	2
Procedures	5	0
Prior work experience	3	3
Faculty mentors^a^	3	1
Clinical exposure MS1/MS2^a^	2	0
EMIG workshops^a^	1	0
“Less competitive specialty”	1	0

^a^ Modifiable factor that can be influenced at the institutional level.

Regarding the time of first exposure, 45.8% of respondents (*n* = 11) were first exposed to EM prior to medical school. 37.5% (*n* = 9) were first exposed during preclinical years, 12.5% (*n* = 3) were first exposed during the third year, and 4.2% (*n* = 1) were exposed during the fourth year.

Regarding timing of their decision to choose EM, 65% of respondents (*n* = 15) decided during the third year, while 17% (*n* = 4) decided in the fourth year, 4% (*n* = 1) in the second year, and 13% (*n* = 3) had decided before medical school.

## 4 Discussion

Our survey asked students to rate the usefulness of various resources for learning about EM and deciding to pursue an EM career. Distinguishing between the two enabled us to assess the benefit of specific resources often utilized by medical schools for each. However, responses overall were similar, which may suggest that the distinction may not be particularly relevant or that there may be substantial overlap in experiences making it difficult for students to distinguish. This lack of distinction may be due to sample size, the respondents' interpretation of the questions, or that the concepts of learning about and choosing a specialty are highly correlated and easily conflated.

For learning about EM, personalized mentoring from residents was more impactful than from attendings. Possible explanations for this include residents' closer proximity to career stage, more interactions with students compared to attendings, and the perception that residents are more approachable.

Although not specifically examined, the RSEC program at our institution may have contributed to the strong influence of resident advising and may suggest one area in which medical schools could invest resources to help augment resident involvement in student education.

Clinical exposure during the third and fourth years, as well as individual advising, was the most influential factor for both learning about EM and choosing it as a specialty. However, one subtle difference emerged: while students reported that learning about EM was most influenced by residents, choosing EM as a career was shaped by interactions with both residents and attendings. Additional factors that influenced the decision to choose EM included exposure earlier in medical school and resident‐led teaching. Notably, preclinical experiences such as those provided by the EMIG were not identified as helpful in learning about EM. Two of the most influential factors, clinical exposure and individual advising, are factors that can be modified at the medical school level and can be implemented at other institutions.

The impact of clinical clerkships on students' perception of EM has evolved. While early literature found that clerkships did not have significant influence on students' perception of EM [17] or correlate with career choice [18], recent studies found that clinical experience and EM clerkships positively impact students' perceptions of EM and increase interest in EM as a career [13, 19]. One study found that providing this later min medical school may be less beneficial [17] which is not consistent with our results. This highlights the growing importance of structured and immersive clinical experiences in shaping students' perceptions and career choices.

While the majority of participants were exposed to EM before third year, 65% made their decision to pursue EM during third year. This suggests that while early exposure can be useful in learning about EM, the decision to pursue it is often finalized after intensive clinical experiences. This reflects the importance of a dedicated third year EM clerkship, which only 65% of institutions offer and only 33% require [17]. Increasing clinical exposure to EM is one potential modifiable area to augment student interest.

Opportunities traditionally utilized by medical schools such as interest groups and sponsored panels or events were less useful for learning about EM and were among the least cited modifiable factors in students' decisions to pursue EM.

## 5 Limitations

While the response rate was 76%, this study is limited by the small number of participants from a single institution. Additionally, the findings may stem partly from experiences unique to our institution and may not be generalizable. The cross‐sectional design may not have captured student influences over time.

There is also potential for recall bias, especially for students removed from the match for 2 years. Additionally, our study covered a period during which we saw both an increase and then subsequent decrease in applicants at our institution, though both years were still above the national average, and it is unclear whether certain factors may have contributed more to either of these trends.

## 6 Conclusions

This study identifies factors that influenced medical students at one institution to choose EM during a period of declining specialty interest. Emphasizing clinical exposure has been previously described as a method to increase student interest in EM, and our results are consistent with this. We also found that individualized mentorship, particularly from EM residents, seems to be a novel strategy to increase student interest and counter the national trend. Future studies across multiple institutions, specifically qualitative work, would help further elucidate the factors influencing medical student career choices. Prospectively studying the impact of establishing a third‐year clerkship on students choosing EM would also be valuable.

## Author Contributions

**Danielle Kerrigan:** conceptualization; methodology; writing – original draft; writing – review and editing; formal analysis. **Anita Knopov:** conceptualization; methodology; writing – original draft; writing – review and editing; formal analysis. **Kaitlin Lipner:** conceptualization; methodology; writing – original draft; writing – review and editing; formal analysis. **Lauren Trembley:** conceptualization; methodology; writing – original draft; writing – review and editing; formal analysis; data curation. **Amy Mariorenzi:** conceptualization; methodology; writing – original draft; writing – review and editing; formal analysis; Data curation. **Brian Clyne:** writing – original draft; writing – review and editing; supervision; conceptualization; methodology; data curation; formal analysis.

## Conflicts of Interest

The authors declare no conflicts of interest.

## Data Availability

The data that support the findings of this study are available from the corresponding author upon reasonable request.

## Appendix A Survey

How useful were the following resources in learning about emergency medicine as a specialty?

**Table:**

	Not at all useful	Somewhat useful	Moderately useful	Very useful	Did not use this resource
Advising/mentoring from attending physicians					
Advising/mentoring from residents					
School‐sponsored career planning workshops and courses					
EM‐sponsored career planning workshops, meetings, or lectures					
Exposure to EM via the interest group					
Participation in clinical electives					

How influential were the following in helping you choose Emergency Medicine?

**Table:**

	Not at all influential	Somewhat influential	Moderately influential	Very influential	Did not use this resource
Advising/mentoring from attending physicians					
Advising/mentoring from residents					
Exposure to EM during 1st or 2nd year of medical school					
Exposure to EM during 3rd year clerkship					
Exposure to EM during 3rd year EM Elective (Advanced Clinical Mentorship)					
Exposure to EM during 4th year clerkship or Sub‐I					
Exposure to EM prior to starting medical school (i.e., work as an EMT, volunteering in an ED)					
Research opportunities within EM					
Resident‐led teaching (lecture or bedside)					
Faculty‐led teaching (lecture or bedside)					
Personal experiences with either you or a friend/family member receiving emergency care					

Of all the factors you considered while choosing a specialty, what were your top 3 most influential factors or experiences that impacted your ultimate choice to pursue EM?

Of your top 3 influential factors or experiences, which is the single most impactful and why was it so impactful?

When was your first exposure to Emergency Medicine?

When did you decide to pursue Emergency Medicine?

What other specialties did you consider and ultimately why did you decide on Emergency Medicine instead?

What impact, if any, has your experience with Emergency Medicine at Brown had on your decision to pursue Emergency Medicine?
